# Progressive painful skin lesions in a patient with end-stage renal disease

**DOI:** 10.1016/j.jdcr.2025.09.041

**Published:** 2025-10-24

**Authors:** Phuong Daniels, Lauren James, Kaitlyn Stocks, Kayd Pulsipher, Wanli Cheng, Ashley Rice

**Affiliations:** a Department of Dermatology, Campbell University School of Osteopathic Medicine, Buies Creek, North Carolina; b Department of Dermatology, Sampson Regional Medical Center, Clinton, North Carolina; c Novant Health - Pathology Department, New Hanover Regional Medical Center, Wilmington, North Carolina

**Keywords:** Calciphylaxis, calcium-phosphate balance, ESRD (end stage renal disease), high mortality, microvascular calcification, painful skin ulcers, sodium thiosulfate

## Case description

**Fig 1:**
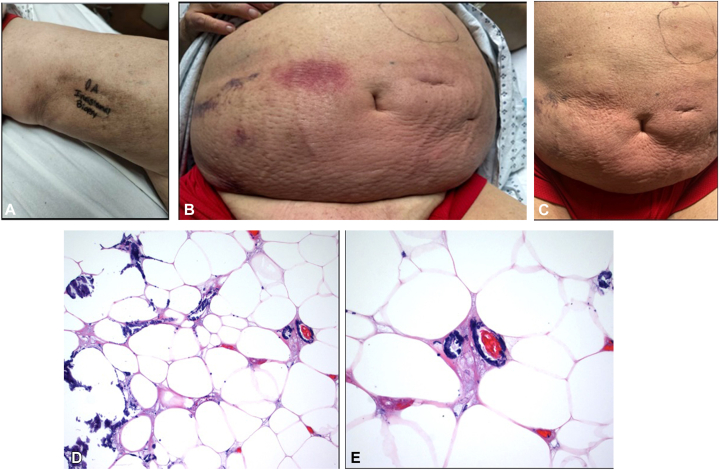
**A,** Hyperpigmented indurated plaque located on the left medial thigh. **B,** Erythematous indurated plaques with a peau d’orange appearance and purpura involving the lower abdomen. **C,** Erythematous indurated plaques with a peau d’orange appearance on the lower abdomen. **D, E,** Showing calcification within the small vessels of the subcutaneous tissue, accompanied by thrombosis and focal tissue necrosis (hematoxylin-eosin stain; original magnifications: **D,** ×20; **E,** ×40).

A 60-year-old male with past medical history of end-stage renal failure on dialysis for 4 years, heart failure with preserved ejection fraction, type 2 diabetes, and gout presented with a 6-week history of progressively painful cutaneous lesions. Physical exam revealed purpura and ill-defined, indurated erythematous plaques with a peau d’orange appearance involving the abdomen (Fig 1, *B* and *C*) and left medial thigh (Fig 1, *A*). Laboratory evaluation demonstrated hypocalcemia (8.5 mg/dL), hyperphosphatemia (8.4 mg/dL), and a markedly elevated parathyroid hormone (PTH) level of 1695.8 pg/mL. Histopathological examination of an initial abdominal punch biopsy revealed mild perivascular lymphocytic infiltration with histiocytes. A subsequent incisional biopsy from the left medial thigh demonstrated calcification within the small vessels of the subcutaneous tissue, accompanied by thrombosis and focal tissue necrosis (Fig 1, *D* and *E*).

**Question 1: Which of the following is the most appropriate next step in management to address the underlying pathophysiology of this patient’s cutaneous findings?**

**A.** High-dose systemic corticosteroids
**B.** Initiate sodium thiosulfate therapy and optimize calcium-phosphate balance
**C.** Empiric broad-spectrum intravenous antibiotics
**D.** Surgical debridement of necrotic tissue
**E.** Increase dietary calcium intake

## Case discussion

**B. Initiate sodium thiosulfate therapy and optimize calcium-phosphate balance – Correct.** This 60-year-old male with end-stage renal disease on dialysis, multiple comorbidities, and markedly elevated parathyroid hormone levels presented with progressively painful plaques and ulcerations. Histopathologic examination demonstrated vascular calcification and thrombosis, confirming a diagnosis of calciphylaxis, a rare condition defined by microvascular calcification and thrombosis that results in painful, ischemic skin lesions.^1^

Key risk factors for calciphylaxis include chronic kidney disease with prolonged dialysis vintage, with the incidence ranging from 0.04% to 4% in dialysis patients.^2^ Less common risk factors include female sex, obesity, hyperparathyroidism, use of calcium-based phosphate binders, and warfarin therapy.^3^ Patients often present with indurated plaques that progress to necrotic ulcers. The cutaneous findings most commonly occur in adipose-rich areas such as the abdomen and thighs and are associated with severe pain due to calcium being deposited in tissues. tissue ischemia. This results in progressive tissue necrosis, which, by the time clinical signs become apparent, signifies that diagnosis is primarily clinical and often requires a high index of suspicion given the patient’s history and cutaneous findings. Skin biopsy can confirm the diagnosis by demonstrating calcification of small dermal and subcutaneous vessels, fibrointimal hyperplasia, and thrombosis.^4^ However, biopsy carries a risk of poor wound healing and infection and is often reserved for atypical presentations or non-ESRD patients. Additional proposed approaches for evaluating calciphylaxis include imaging, laboratory evaluation (metabolic panel, hepatic function, coagulation studies, and fetuin-A), and nuclear bone, although these are less sensitive than histological evaluation.^2^

Treatment often includes a multidisciplinary approach that and focuses on risk factor modification aimed at optimization of calcium-phosphate balance (ie, correction of hypercalcemia/hyperphosphatemia, discontinuation of vitamin D, calcium-based binders, and warfarin), intensified dialysis, aggressive wound care, pain management, and infection control. Sodium thiosulfate (intravenous 12.5-25 g 3 times weekly) is commonly used off-label for its potential to dissolve tissue calcium deposits.^4^^,^^2^ Cinacalcet may also be considered for hyperparathyroidism, and parathyroidectomy is reserved for refractory cases.^1^ Multimodal therapy is associated with improved outcomes, but resolution is uncommon and mortality often remains high. Studies indicate a mortality rate of 60% to 80%, with sepsis secondary to nonhealing ulcers being the leading cause of death.^5^

## Conflicts of interest

None disclosed.
